# Microbiome or Infections: Amyloid-Containing Biofilms as a Trigger for Complex Human Diseases

**DOI:** 10.3389/fimmu.2021.638867

**Published:** 2021-02-26

**Authors:** Amanda L. Miller, Shingo Bessho, Kaitlyn Grando, Çagla Tükel

**Affiliations:** Department of Microbiology and Immunology, Lewis Katz School of Medicine, Temple University, Philadelphia, PA, United States

**Keywords:** curli, biofilm, microbiome, systemic lupus erythematosus, reactive arthritis, Parkinson's disease, Alzheimer's disease, colorectal cancer

## Abstract

The human microbiota is the community of microorganisms that live upon or within their human host. The microbiota consists of various microorganisms including bacteria, fungi, viruses, and archaea; the gut microbiota is comprised mostly of bacteria. Many bacterial species within the gut microbiome grow as biofilms, which are multicellular communities embedded in an extracellular matrix. Studies have shown that the relative abundances of bacterial species, and therefore biofilms and bacterial byproducts, change during progression of a variety of human diseases including gastrointestinal, autoimmune, neurodegenerative, and cancer. Studies have shown the location and proximity of the biofilms within the gastrointestinal tract might impact disease outcome. Gram-negative enteric bacteria secrete the amyloid curli, which makes up as much as 85% of the extracellular matrix of enteric biofilms. Curli mediates cell-cell attachment and attachment to various surfaces including extracellular matrix components such as fibronectin and laminin. Structurally, curli is strikingly similar to pathological and immunomodulatory human amyloids such as amyloid-β, which has been implicated in Alzheimer's disease, α-synuclein, which is involved in Parkinson's disease, and serum amyloid A, which is secreted during the acute phase of inflammation. The immune system recognizes both bacterial amyloid curli and human amyloids utilizing the same receptors, so curli also induces inflammation. Moreover, recent work indicates that curli can participate in the self-assembly process of pathological human amyloids. Curli is found within biofilms of commensal enteric bacteria as well as invasive pathogens; therefore, evidence suggests that curli contributes to complex human diseases. In this review, we summarize the recent findings on how bacterial biofilms containing curli participate in the pathological and immunological processes in gastrointestinal diseases, systemic autoimmune diseases, and neurodegenerative diseases.

## Introduction

The community of microorganisms that live upon or within a host are referred to as the microbiota. The human microbiota includes bacteria, fungi, viruses, and archaea that colonize the surface or deep layers of the skin (skin microbiota), the mouth (oral microbiota), the vagina (vaginal microbiota), and the digestive tract (gut microbiota) (1). The human microbiota has received increasing attention in numerous research fields over the last 15 years. The gut microbiota is of interest as numerous studies have reported that there are changes in the gut microbiota during obesity, diabetes, liver diseases, cancer, and neurodegenerative diseases (2–7).

Studies of the diversity of the human microbiota started as early as the mid-seventeenth century with Antonie van Leeuwenhoek who compared the oral and the fecal microbiota. He observed differences between the microbes in these two locations and also between samples from healthy vs. diseased individuals (8). In 2001, Joshua Lederberg coined the term microbiome to refer to the “the ecological community of commensal, symbiotic, and pathogenic microorganisms that literally share our body space” (9). Many fundamental questions concerning the human microbiota have been difficult or impossible to address until recently. In 2017, ~4,000 publications focusing on the study of the gut microbiota were published, accounting for 80% of the publications on the subject since 1977 (2). The advancement of laboratory techniques and “omics” technologies have allowed researchers to characterize the composition of the microbiota and its functions in human health and disease (10).

The coevolution of humans and their microbial symbionts have selected for a specialized community of microorganisms that thrive in the gut (11). Bacteria comprise the bulk of the gut microbiota with archaea, eukaryotes, and viruses present in much smaller numbers (10). In a healthy human adult, the gut microbiota is dominated by two phyla: Firmicutes and Bacteroidetes (12). Other phyla, including Actinobacteria, Proteobacteria, Verrucomicrobia, and Euryarchaeota, are found in lower abundance (12). The gut microbiota is unlike any free-living microbial communities found in the environment due to the unique environment of the digestive tract (13). The microbiota plays a crucial role in maintaining immune and metabolic homeostasis and protecting the host against pathogens through microbial crosstalk with the mucosal immune system through integrated signaling pathways and gene regulatory networks (13–15). The interactions between the host immune system and the colonizing gut microbiota initiate at birth and are important for host immune system development and homeostasis (16–18). A variety of genetic and environmental factors influence the composition and the function of the gut microbiota including host diet, genetics, age, location, and medication use, especially antibiotics (19). When this homeostatic relationship is disrupted, it can lead to dysbiosis or “an imbalance in the composition and metabolic capacity of our microbiota” (20). Growing evidence indicates that dysbiosis shifts the microbiota in ways that increase inflammation and accelerate the onset or contribute to the pathogenesis of chronic diseases (20) such as cardiovascular disease, obesity, diabetes, cancer, asthma, and inflammatory bowel disease (20–26).

Many species of bacteria that colonize the gut live in biofilms. A bacterial biofilm is a group of bacteria that are encapsulated in a three-dimensional, self-produced extracellular matrix that is adhered to a biotic or abiotic surface (27, 28). The biofilm provides a layer of protection to microorganisms that grow in stressful environments where nutrients are scarce and during changes in temperature, osmolarity, and oxygen availability (29–31). Furthermore, the biofilm blocks access by toxic agents such as antibiotics and the host's immune system (32).

Biofilms can be composed of a single species of bacteria or a consortium of multiple species of bacteria. Biofilms can be formed by a variety of bacterial species including Gram-positive (e.g., *Bacillus* spp, *Listeria monocytogenes, Staphylococcus* spp, and lactic acid bacteria including *Lactobacillus plantarum* and *Lactococcus lactis*) and Gram-negative species (e.g., *Escherichia coli, Salmonella enterica*, and *Pseudomonas aeruginosa*) (33). The Proteobacteria phylum, which makes up ~0.1% of the gut microbiota in a healthy individual, expands during inflammation. The Gammaproteobacteria class includes several medically and scientifically important families including *Enterobacteriaceae*, Vibrionaceae, and Pseudomonadaceae. *Enterobacteriaceae* is a large family of Gram-negative bacteria that includes many harmless enteric commensals as well as pathogens such as *Salmonella, E. coli, Yersinia pestis, Klebsiella*, and *Shigella*. All these bacteria have the ability to make biofilms (30, 34–37).

Bacterial biofilms form throughout the human oro-gastrointestinal tract and mixed species biofilms have been observed in dental and gastric infections as well as in intestinal diseases, chronic gut wounds, and colon cancer (38). Biofilms are also formed by species necessary for a healthy gut mucosa, and these biofilms may benefit the host by fortifying host defenses, enhancing the exchange of nutrients between the microbiota and the host, and interfering with colonization by pathogenic bacteria (38). Therefore, biofilms within the human gut can be both beneficial and detrimental to the host depending on whether they are produced by the commensal microbiota or enteric pathogens (38).

Only 10% of the biomass of the biofilm are actual bacterial cells (29, 39). The composition and the structure of the biofilm is dependent upon the bacteria within it and the environment in which it is formed (27, 28). The bacterial cells within a biofilm are physiologically distinct from their planktonic counterparts (40). In a biofilm, cells are embedded within an extracellular matrix (ECM) composed of extracellular polymeric substances such as lipids, polysaccharides, proteins, and DNA (41–43). The ECM accounts for 90% of the total biomass of the biofilm. In *E. coli* biofilms, the major proteinaceous component, which comprises 85% of the ECM, is the amyloid curli. Curli encapsulates individual bacterial cells and forms an interwoven mesh that supports the ECM (44–46). Curli expression is triggered when enteric bacteria are grown under stressful environmental conditions that favor biofilm formation over planktonic cell growth. Curli is responsible for the overall development of the biofilm architecture (47–50) as curli-deficient bacteria do not form mature three-dimensional biofilms and only grow in a single cell layer.

Curli proteins form thin amyloid fibers on the surface of enteric bacterial cells (51–53). These fibers range from 4 to 10 nm in width and have a β-sheet-rich structure in which the β-sheet strands are orientated perpendicular to the axis of the fiber (54). Human amyloids also have a cross-beta structure and share a strikingly similar quaternary structure with bacterial amyloids, including the pathological and immunomodulatory human amyloids such as amyloid-β (Aβ), which is involved in Alzheimer's disease (AD), α-synuclein (αSyn), which is implicated in Parkinson's disease (PD), and serum amyloid A (55–59).

Like other amyloids, curli is a conserved molecular pattern that causes the activation of toll-like receptors (TLR) 1 and 2 as well as intracellular NLR Family Pyrin Domain Containing 3 (NLRP3) inflammasome (60–62). Studies have shown that the immune system recognizes both bacterial amyloid curli and human amyloids through the same receptors that facilitate the inflammatory processes (61). Recent studies have demonstrated that curli and curli-associated biofilms in the gut participate in the pathogenesis of human diseases, including colorectal cancer, systemic lupus erythematosus (SLE), and PD (Table 1). In this review, we summarize the recent findings that suggest how bacterial biofilms containing curli participate in pathological and immunological processes of these diseases by direct interactions such as cross-seeding of human amyloids and by indirect interactions that trigger inflammation.

**Table 1:** Human diseases exaggerated by bacterial biofilms or biofilm by-products.

**Biofilm related condition/disease**	**Bacteria**	**Biofilm component/byproduct**	**Proposed mechanism**
Systemic lupus erythematosus (SLE)	*S*. Typhimurium*, E. coli*	Amyloid curli, other bacterial amyloids	Formation of complexes between curli and DNA increases type I interferons and autoantibody production leading to disease flares
Reactive Arthritis (ReA)	*S*. Typhimurium,	Amyloid curli	Increases proinflammatory cytokines and autoimmune response leading to joint inflammation
Parkinson's disease and Alzheimer's disease	*E. coli, Pseudomonas*	Amyloid curli, FapC	Neuroinflammation, increasing fibrillation and deposition of α-synuclein or amyloid-β in the brain
	Commensal Gram-negative bacteria	Endotoxin	During aging, increases blood-brain-barrier permeability, co-localizes with Aβ plaques in the brain, implicated in aggregation of α-synuclein
	*Porphyromonas gingivalis*	Gingipains	Found within the brains of AD patients, associated with neurotoxicity and neuroinflammation
Colorectal cancer	Fusobacteria, *Alistipes*, Porphyromonadaceae, Coriobacteridae, *Staphylococcaceae, Akkermansia*, Methanobacteriale	Unknown	Significant outgrowth of these bacteria in intestinal microbiota of CRC patients
	*Bacteroides fragilis, E. coli*	*B. fragilis* toxin, colibactin and biofilms	Carcinogenic toxins and biofilms propagate the formation of tumors
	*Fusobacterium nucleatum*	Adhesin molecule FadA	Induces oncogenic and inflammatory responses in the gut
	*Campylobacter jejuni*	Cytolethal descending toxin	Induces changes in microbial composition and toxigenic processes

## Curli Containing Bacteria in The Gastrointestinal Tract and The Urinary Tract

**Figure 1:**
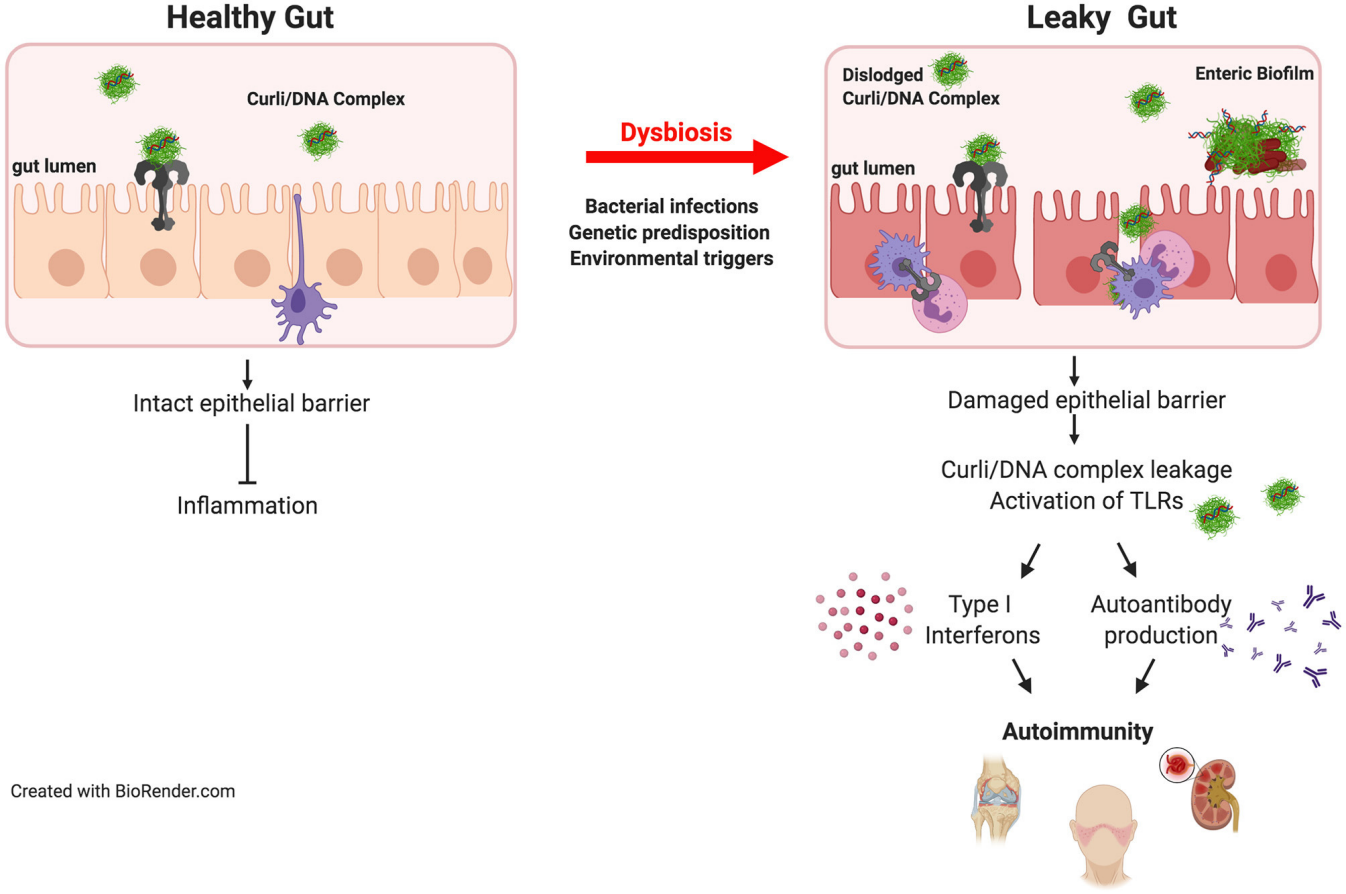
Bacterial amyloids and autoimmunity. Amyloids are produced by the members of gut microbiome. Curli/DNA complexes produced by commensal bacteria are recognized by TLR2/TLR1 heterocomplex which dampens inflammation in healthy intestinal tract. However, when the epithelial barrier is damaged during invasive infections or by other environmental factors or diseases, dislodged curli/DNA complexes from the biofilm activates TLR2/TLR1 heterocomplex and TLR9 leading to the generation of type I interferons and autoantibodies resulting in initiation and exacerbation of autoimmunity. In genetically predisposed individuals, the autoimmune effects of the curli/DNA complexes will likely to be exacerbated.

Biofilms can occur throughout the entire length of the gastrointestinal tract (38) and can be disease-linked or important for health. Two driver species of pathogenic biofilm formation in the gut are *Salmonella* and *E. coli*. These enteric bacteria thrive in a pro-inflammatory environment, conditions in which they outcompete the commensal microbiota. An outgrowth of enteric bacteria is common during inflammatory states that are associated with many gut disorders (63). It was highly debated whether or not enteric bacteria expressed curli and grow as biofilms in the gut. However, there was indirect evidence in support of this: for example, patients recovering from *E. coli*-induced sepsis harbor antibodies against curli (64). Similarly, antibodies against curli are detected after infection of mice with *Salmonella enterica* serovar Typhimurium (65). A recent study showed direct evidence for curli synthesis in the intestinal tract by *S*. Typhimurium during infection (66). Curli is recognized as a PAMP by the mucosal immune system *via* TLR2/TLR1 heterocomplex (60, 62) leading to the activation of *NF-*κ*B*, eliciting the production of proinflammatory chemokines and cytokines including, IL-6, IL-8, and IL-17A (61, 67). The detection of curli by a healthy gut mucosa leads to reinforcement of the gut barrier preventing the leakage of bacteria and possibly the pathological bacterial amyloids (Figure 1) (68, 69).

The urinary tract microbiome is just beginning to be characterized. Uropathogenic *E. coli* (UPEC), the most common cause of urinary tract infections (UTIs) (70, 71), is a member of the family *Enterobacteriaceae* and has the ability to form a curli-containing biofilm (72). UPEC is a frequent colonizer of medical devices and the primary cause of recurrent urogenital infections (73). UPEC forms curli-containing biofilms that are difficult to treat and eradicate with antibiotics leading to multidrug resistance (73). UTI infections can become persistent and result in bacteriuria which can lead to sepsis (71). Expression of *csg* gene cluster, genes that encode curli, is upregulated in UPEC isolated from urine from patients suffering from UTIs, whereas no *csg* expression is detected in urine from healthy controls (74, 75). It was reported that curli promotes colonization and immune induction by enhancing bacterial adherence and invasion into the uroepithelium during early stages of UTI (74).

## The Involvement of Bacterial Amyloids in Autoimmune Diseases

The primary function of the immune system is to protect the host from possibly harmful substances and pathogens. The first line of defense against non-self-pathogens is the innate immune response. It immediately prevents the spread of foreign pathogens throughout the body. The second line of defense is the adaptive immune response, which is specific to the pathogen presented. This response is long lasting and highly specific. The exact etiologies for autoimmune disorders such as rheumatoid arthritis, SLE, and inflammatory bowel disease remain unknown, but various genetic and environmental factors contribute to their development (76). Furthermore, in those individuals who are predisposed, self-tolerance, the ability of the immune system recognizing self-produced antigens as non-threatening, becomes disrupted. The immune system begins to recognize self-antigens as foreign, leading to the bodies inability to “tolerate” self tissues as it attacks itself causing systemic and organ-specific damage (76–78). Numerous microorganisms use molecular mimicry or mimotopes to avoid detection by the immune system, which in turn could amplify the autoimmune response.

Infections and exposure to pathogens or opportunistic organisms may initiate or exacerbate autoimmune disorders. In addition to true autoimmune diseases mentioned above, a small group of patients experience autoimmune symptoms months and sometimes years after an infection is cleared. These autoimmune sequelae are observed following infections with human pathogens such as *E. coli, Borrelia burgdorferi, S*. Typhimurium, *Mycobacterium tuberculosis, P. aeruginosa*, Group A streptococci, and *Staphylococcus aureus* (76, 79–87). Joints are affected in many cases and post-infectious arthritis is observed. Most interestingly, all these bacteria express curli-like amyloids and form biofilms during infections. One of best understood examples of post-infectious arthritis is reactive arthritis (ReA). ReA is an inflammatory arthritis that develops in 5–10% of the patients following gastrointestinal infections with *Salmonella, Shigella, Yersinia*, or *Campylobacter* or following genital infections with *Chlamydia trachomatis* (79). Symptoms usually start 1–4 weeks post-infection and can last more than 5 years (79). Histocompatibility leukocyte antigen (HLA) B27 allele is a risk factor for ReA. About 90% of individuals who develop ReA following *Salmonella* infection carry the HLA-B27 genotype (88). ReA patients are not responsive to antibiotic treatment and cultures of joint fluids yield no bacterial growth. However, one study that employed immunohistochemical staining and mass spectrometry reported the presence of bacterial byproducts in synovial fluid (89–91). A recent study showed that in a mouse model of *Salmonella* infection curli is synthesized in the gastrointestinal tract and leads to increased anti-double-stranded DNA autoantibodies and to synoviocyte proliferation coupled with bone resorption in the knee joints (66). Infection with a curli mutant or a non-invasive strain did not cause such responses suggesting that the presence of curli during invasive infection with *S*. Typhimurium causes ReA (66).

Several mechanisms have been suggested to underlie the development of ReA. T cell-mediated immune responses clearly play a large role in autoimmune diseases. In rheumatoid arthritis, another autoimmune disease that affects joints, the functions of certain subsets of CD4^+^ T cells with regulatory capacity such as CD25^+^ regulatory T cells and Th2 cells are severely impaired. As ReA is a rare condition the role of these cells have not been elucidated, but it was proposed that CD4^+^ T cells that produce IL-17 and generate a type 17-mediated inflammatory response contribute to joint damage (92). Curli binds to and activates TLR2, leading to the production of pro-inflammatory cytokines and chemokines including IL-6, IL-8, TNFα, and IL-17 (60, 61, 67, 93). Therefore, TLR2-mediated IL-17 production is a plausible mechanism for the curli-driven development of ReA. However, recent studies have also shown that curli binds to DNA in the ECM forming highly immunogenic curli/DNA complexes. Curli amyloid acts as a carrier to bring DNA into endosomes where the DNA is recognized by TLR9 and activates type I interferons (94). Curli/DNA complexes also trigger the generation of anti-dsDNA and anti-chromatin autoantibodies following translocation into systemic sites from the gut (Figure 1) (41, 66, 94). In autoimmune diseases where anti-dsDNA autoantibodies are observed, DNA seems to be the key component. Nevertheless, it is not known whether the autoantibodies generated during *S*. Typhimurium infection that recognize curli alone or curli/DNA complexes facilitate joint damage directly. Additional studies are needed to assess the role of anti-dsDNA autoantibodies and to determine whether curli without DNA can elicit joint inflammation and damage.

As curli is also be produced commensal strains from the *Enterobacteriaceae* family (69), dissecting the mechanisms by which curli-producing bacteria trigger arthritogenic processes in autoimmune diseases is critical. Studies have shown that bacteria from the normal gut microbiota can also be arthritogenic and cause experimental arthritis in animals (95). Antibiotic treatment can prevent and suppress arthritis in murine models prone to arthritis. Additionally, germ-free animals do not develop arthritis (95). Overall, these studies suggest that amyloids from the gut microbiota contribute to the autoimmune processes. Phylogenetic analysis suggests that the curli assembly machinery is widespread, as homologs of the *csg* genes, which encode curli and are responsible for its biosynthesis and secretion, are found within four phylum, Bacteroidetes, Proteobacteria, Firmicutes, and Thermodesulfobacteria (30, 96, 97). As these members of these bacterial phyla are found in the gut microbiota and biofilms are observed in the intestinal tract, it is likely that the gut microbiota harbors amyloids.

SLE is a classical autoimmune disease in which the immune system causes widespread inflammation and tissue damage in joints, skin, brain, lungs, kidneys, and blood vessels. Bacterial infections are a major cause of morbidity and mortality in patients leading to and exacerbating SLE flares. Epidemiological studies suggest that bacterial infections promote SLE disease in predisposed individuals, but the underlying mechanisms remain unknown. SLE patients are more susceptible to infections, particularly bacterial infections, involving the upper respiratory tract, skin, and urinary tract than subjects without SLE (98–101). SLE patients produce autoantibodies against a wide variety of cellular antigens including double-stranded DNA and nuclear proteins (41). Curli/DNA complexes are powerful immune stimulators (41). When given systemically, curli/DNA complexes trigger immune activation and production of type I interferons as well as autoantibodies in SLE-prone and wild-type mice (41). It was also found that infection of SLE-prone mice with curli-producing bacteria triggers higher autoantibody titers than do curli-deficient bacteria (41). Furthermore, clinical studies of SLE patients with persistent bacteriuria and *E. coli* within their urine were positive for anti-curli/DNA antibodies (102). The levels of anti-curli/DNA IgG correlated with both bacteriuria and flares in the SLE cohort, further suggesting a link between curli/DNA complexes and increased disease severity in SLE (102). These data suggest that enteric bacteria production of curli potentiates disease pathogenesis in individuals predisposed to autoimmune disorders.

## Neurological Diseases

**Figure 2:**
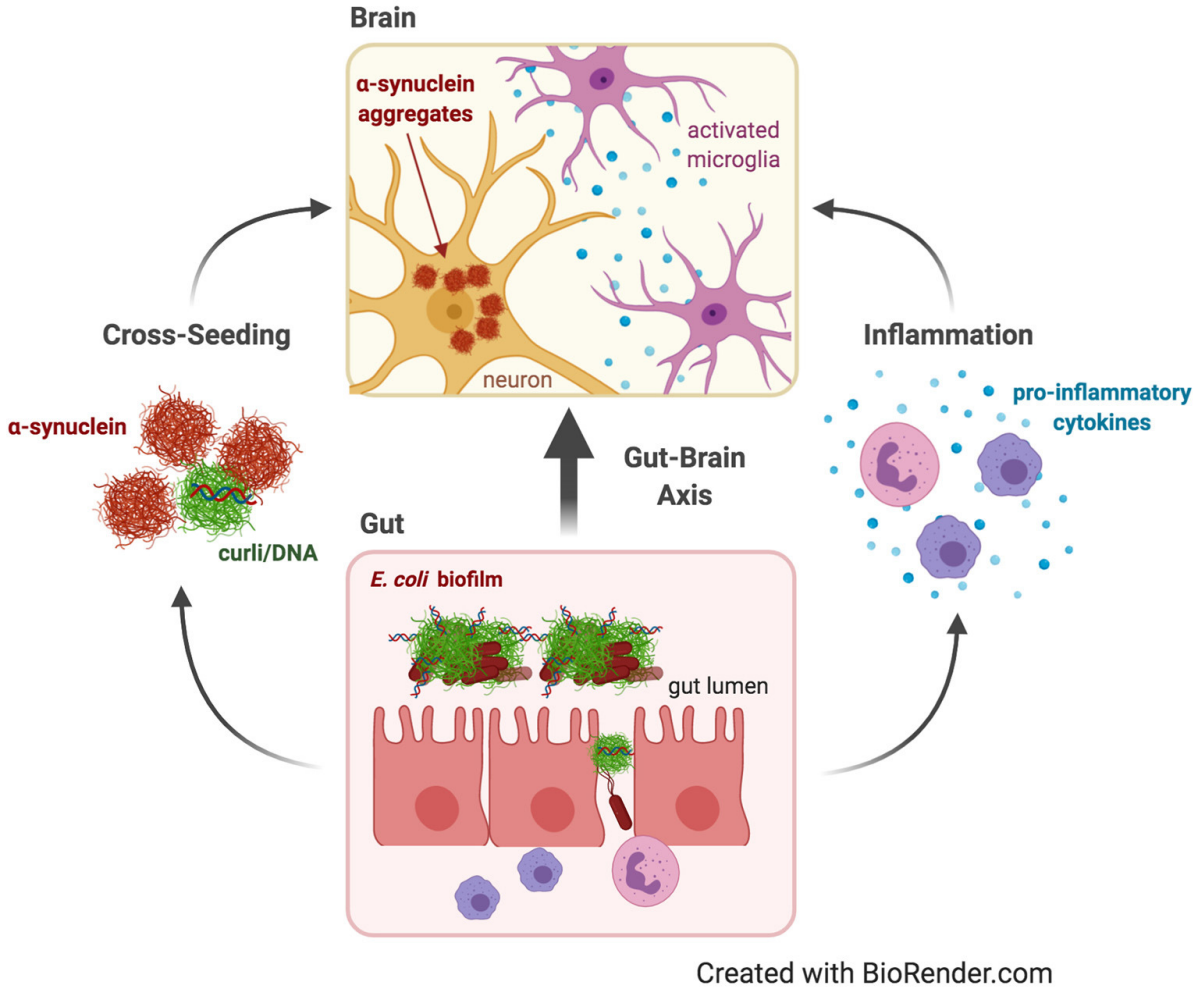
Parkinson's disease. Dysbiosis and leaky gut often occur with age, allowing contents of the gut lumen, such as curli from *E. coli* biofilms, to escape and induce changes in the brain through the gut-brain axis. Curli may contribute to Parkinson's Disease by increasing systemic inflammation and neuroinflammation in the form of activated microglia and astrocytes and elevated pro-inflammatory cytokines. Curli may also cross-seed fibrillation and aggregation of α-synuclein, which is capable of prion-like propagation from the gut to the brain to cause neurodegeneration.

Neurodegenerative diseases such as Alzheimer's Disease (AD) and Parkinson's Disease (PD) are characterized by proteins such as Aβ, hyperphosphorylated tau, and αSyn misfolded into pathological amyloid aggregates in and around neurons; these aggregates are associated with elevated inflammation (103–105). The gut microbiota impacts the nervous system through the gut-brain axis, a bidirectional “highway” for immune, metabolic, endocrine, and neural signals (Figure 2) (106, 107). Gut bacteria can synthesize neurotransmitters and bacterial metabolites, like indoles and short-chain fatty acids that can bypass the blood-brain-barrier and impact the brain (108–110). One hypothesis is that these signals and microbial products are transmitted not through the circulatory system, where they would need to navigate the blood-brain-barrier, but *via* the vagus nerve: the longest cranial nerve in the body, connecting the enteric nervous system to the brain stem, and containing both afferent and efferent fibers (111–113). Thus, it is similarly possible that aggregation of human amyloidogenic proteins may be seeded or indirectly induced by bacterial amyloids that originate in the gut.

There are two predominant theories about how biofilm-forming gut bacteria might affect neurodegenerative disease: indirectly by provoking neuroinflammation or directly due to cross-seeding of aggregation of human amyloids by bacterial amyloids (Figure 2). Neuroinflammation is emerging as a critical component of neurodegenerative diseases. It involves chronic activation of microglia and astrocytes, elevated pro-inflammatory cytokines and chemokines, and accumulation of Aβ and αSyn (104, 105). Dysbiosis of the gut microbiota may cause neuroinflammation by increasing pro-inflammatory cytokines, systemic inflammation, and weakening of the gut barrier by decreasing immune regulatory function (114, 115). Aging is a major risk factor for neurodegenerative diseases, and aging has been shown to change the microbiome composition and increase inflammation (116, 117). Aging also disrupts blood-brain-barrier function, often as a result of dysbiosis, allowing passage into the brain of bacterial cells or inflammatory metabolites that may exacerbate neuroinflammation (118–120).

Bacterial amyloids such as curli share structural and physical properties with human pathogenic amyloids (76, 121). Curli activates the same TLRs that recognize Aβ and αSyn (62, 122, 123). Curli induces elevated pro-inflammatory cytokines such as TNF-α, IL-6, and IL-1β, which have been shown to impair blood-brain-barrier integrity (64, 124–126). Therefore, it has been proposed that bacterial amyloids cause neuroinflammation and induce protein aggregation, indirectly leading to neurodegeneration (123, 127, 128). In addition, Aβ, tau, and αSyn have antimicrobial properties and so aggregation may be triggered by inflammation and dysbiosis (129–133).

PD is characterized by a loss of dopaminergic neurons in the nigrostriatal pathway. This loss is caused by Lewy bodies and Lewy neurites composed of intraneuronal αSyn aggregates (Figure 2) (103). Braak hypothesized that PD begins in the enteric nervous system and spreads up the vagus nerve to the brain (134). The earliest symptoms of PD, which occur up to 20 years before diagnosis in more than 65% of patients, include delayed gastric emptying, gastroparesis, constipation, and other gastrointestinal dysfunctions (135, 136). PD patients were recently demonstrated to have an altered gut microbiome compared to healthy controls (5). Elimination of gut bacteria in transgenic mouse models expressing human αSyn, either by using germ-free mice or treating with antibiotics, resulted in decreased αSyn deposition, neuroinflammation, and motor deficits; an effect that was reversed by recolonizing mice with microbiota from PD patients but not with healthy human microbiota (137). Furthermore, colonization of Fischer 344 rats with *E. coli* led to increased αSyn deposition in gut and brain neurons, increased gliosis, and increased inflammatory cytokines; effects replicated in *C. elegans*, but not observed with *E. coli* incapable of producing curli (Table 1) (127). A recent study corroborated these results: colonization of αSyn-overexpressing mice with wild-type *E. coli*, and not with curli-deficient *E. coli*, resulted in elevated αSyn pathology in the gut and brain and exacerbated cognitive, intestinal, and motor deficits and increased neuroinflammation. Interestingly, treatment of these mice with epigallocatechin gallate, an amyloid inhibitor restricted to the gut, prevented the increase in PD pathology and behavioral deficits. The same study confirmed *in vitro* that purified curli accelerated αSyn aggregation (138). Additionally, FapC, a functional amyloid produced by *Pseudomonas* biofilms, contributed to αSyn fibrillation, whereas a mutated FapC inhibited αSyn fibrillation (Table 1) (139). Thus, these studies also support the second theory: that bacterial amyloids directly cross-seed the aggregation of human pathological amyloids in the gut, which spread to the brain.

Human amyloids Aβ and αSyn have proven capable of cross-seeding tau (140, 141). Curli homologs from different bacteria strains are also capable of cross-seeding, even between *E. coli* and *Shewanella onedensis* (142). The first paper to propose the idea of cross-seeding by non-mammalian proteins showed that *E. coli* curli accelerated the fibrillation of serum amyloid A in a secondary amyloidosis mouse model (143). Data from *in vitro* studies also suggest that cross-seeding of Aβ aggregation by bacterial amyloids could initiate AD (Table 1) (127, 144). Curli is capable of cross-seeding Aβ fibrillation, and treatment of *S*. Typhimurium with D-enantiomeric peptides, known to inhibit Aβ fibrillation, inhibits curli fibrillation and reduces biofilm formation (121). Infection of pulmonary microvascular endothelial cells with clinical *P. aeruginosa*, which produce the bacterial amyloid FapC, induces production of Aβ and tau, capable of prion-like propagation to naïve cells (145). Evidence from animal models will be required to determine whether bacterial amyloids are capable of directly seeding Aβ or tau aggregation in a manner relevant to neurodegeneration.

Numerous recent studies confirmed that αSyn aggregates are capable of propagating from the gut to the brain, causing cognitive and motor deficits. Mice injected with αSyn preformed fibrils into the duodenal or gastric wall developed Lewy body-like αSyn aggregates in the dorsal motor nucleus of the vagus nerve, which spread to the brain and resulted in motor deficits and neurodegeneration (146, 147). The retrograde transport of αSyn pathology to the brain was corroborated in rat models (112, 148). However, studies by one group found that the pathology failed to progress past the dorsal motor nucleus (149, 150). Another group injected baboons with patient-derived Lewy body extracts and found that pathology spread to the central nervous system through circulation rather than *via* the vagus nerve (151). Regardless, there is mounting evidence indicating that bacterial amyloids such as curli, present at high levels in biofilms in the gut, are capable of cross-seeding αSyn aggregation and that αSyn aggregates are capable of spreading from the gut to the brain as a possible initiating event in PD (112, 127, 134, 139, 146–148, 151).

Meanwhile, similar observations are being made in AD, with some important differences from PD. AD is characterized by progressive accumulation of both extracellular Aβ plaques and intraneuronal hyperphosphorylated tau aggregates called neurofibrillary tangles (103). Evidence is inconclusive whether AD pathology begins in the intestines. Some samples from AD patients show increased Aβ deposits in the intestines (152), and several AD mouse models corroborate increased Aβ plaques and hyperphosphorylated tau overexpression in the intestines and enteric nervous system as well as impaired gut motility and function and increased inflammation (152–155). However, one study found no difference in gut motility and absorption between AD and control mice, though they detected intestinal Aβ and tau deposits in AD mice and AD patient samples (156). Another did not find evidence of Aβ in enteric neurons of an AD mouse model (157). Elimination of the gut bacteria by antibiotic treatment of APP/PS1 mice led to reduced Aβ plaque load and associated gliosis, altered cytokine profile, and increased regulatory T cell levels (158, 159). AD patients, and AD mouse models, show altered gut microbiota composition, favoring pro-inflammatory species, compared to healthy patients or wild-type mice (160–162). Microbiome composition affected cognition in APP/PS1 transgenic mice (162). Germ-free APP/PS1 transgenic mice showed reduction in Aβ deposition and pro-inflammatory cytokines in the brain (160). A recent study showed that fecal microbiota transplant from wild-type mice reduced Aβ plaques and tau tangles, gut permeability, systemic and neuroinflammation, and cognitive deficits in an AD mouse model (163). However, there is no *in vivo* evidence directly linking biofilm-forming bacteria and bacterial amyloids in the gut to AD pathology.

Rather than cross-seeding, AD microbiome studies point to a pattern of systemic inflammation and gut leakage that lead to AD pathology and cognitive deficits. Recent studies established that AD neuroinflammation involves NLRP3 inflammasome activation in microglia (164–166). Systemic inflammation induced by endotoxin or by fecal microbiota transplant from AD patients exacerbated microgliosis *via* the NLRP3 inflammasome in a mouse model (167, 168). Interestingly, curli activates the NLRP3 inflammasome in macrophages *in vitro*, suggesting a mechanism of microbiota-induced neuroinflammation (124). The endotoxin hypothesis also posits that microbiota-induced neuroinflammation underlies AD (169). Endotoxin, also known as lipopolysaccharide, produced by Gram-negative bacteria, is elevated in the serum of AD patients and during aging, has been shown to increase blood-brain-barrier permeability, and has even colocalized with Aβ plaques in the brain (Table 1) (169, 170). Endotoxin has also been implicated in the aggregation of a strain of αSyn in PD (171).

Another interesting possibility is direct infection of the brain in AD patients. Some have suggested that there is a separate microbiome within the brain, dominated by proteobacteria as well as fungal species (170, 172–174). If true, this would support the antimicrobial hypothesis of Aβ, wherein Aβ is secreted in response to infection as an antimicrobial peptide (129, 130). Still other studies implicate *Porphyromonas gingivalis*, another biofilm-forming bacteria, which causes chronic periodontitis in the mouth. *P. gingivalis* has been found in the brains of AD patients and is associated with neurotoxicity and neuroinflammation (Table 1) (175). Such studies are controversial due to contamination concerns during autopsy (176) and require further investigation. Regardless of how exactly the microbiome affects AD, antibiotics and probiotics have been proposed as treatment options (177–179).

## Biofilms in Colorectal Cancer and Other Diseases of The Gastrointestinal Tract

Cancer results from uncontrolled, malignant cell proliferation caused by accumulated genetic and epigenetic mutations. The triggers for these mutations are multifactorial in origin and remain elusive in many cases, but genetics play a critical role. Accumulating evidence also supports the involvement of infectious agents in the development of cancer, especially in those organs that are exposed to microorganisms. Approximately 20% of cancers around the world have been estimated to be caused by microbes (180). For example, human papillomaviruses and the bacterium *Helicobacter* pylori cause cervical and gastric cancers, respectively (181, 182). Studies using *Helicobacter* have demonstrated that the protein encoded by cytotoxin-associated gene (*cagA*) induces DNA damage and that host-derived inflammatory mediators and growth factors are direct risk factors for carcinogenesis (182).

Colorectal cancer (CRC) is the fourth leading cause of cancer-related deaths worldwide (183). About 10% of CRC cases are hereditary, and the rest are sporadic. Risk factors include age, genetics, diet, and environmental factors (184, 185). Unhealthy behaviors such as physical inactivity, smoking, consumption of red and processed meat, and alcohol consumption increase risk of CRC (186–189). Some diseases, including obesity and type II diabetes, are also associated with increased risk of CRC development (190). Chronic inflammation is one of the major risks of CRC. Patients with inflammatory bowel diseases, including ulcerative colitis and Crohn's disease, are at risk for development of colitis-associated CRC (191, 192). The susceptibility of animal models of CRC, such as *APC*^*Min*/+^ mice (which carry a germline mutation in *Apc* gene) and azoxymethane-treated mice, is enhanced when dysbiosis is induced by the inflammatory agent dextran sodium sulfate (193, 194).

Further, the intestinal microbiota has emerged as an important factor in CRC initiation and progression. The current view is that CRC initiation is triggered by local mucosal colonization by pathogenic bacteria. Healthy human colon is protected by a mucosal barrier that separates the microbiome from direct contact with the colonic epithelium of the host (195). Reduction of the mucosal barrier increases the contact between the microbiota and colonic epithelial cells and thus constitutes a significant primary step in inciting modifications in the biology of cells and inflammation. Specific changes within the intestinal microbial community are observed in CRC patients, such as increased abundance of Fusobacteria, *Alistipes*, Porphyromonadaceae, Coriobacteridae, Staphylococcaceae, *Akkermansia*, and Methanobacteriales, while representation of *Bifidobacterium, Lactobacillus, Ruminococcus, Faecalibacterium, Roseburia*, and *Treponema* is decreased (196).

Recently, bacterial biofilms were observed in direct contact with CRC tumors upon mucosal degradation. Biofilms exist in the healthy gut mostly within the lumen and away from the epithelium. The fact that the biofilms were in direct contact with the CRC tumors, especially those located in the right colon of humans (determined as proximal host colon to the hepatic flexure) suggests that bacteria or bacterial products are connected to the initiation cell transformation events (197).

Although it was initially thought that biofilms were only on the tumors, patients with biofilm-positive tumors also had biofilms on their tumor-free mucosa. These findings suggest that in patients with biofilm-positive tumors, the luminal environment may provide an ideal landscape for biofilm development. However, the factors that facilitate biofilm development in the colon are difficult to identify as our knowledge of regulatory signals for bacterial biofilm formation is limited. Additionally, presence of bacterial biofilms on tumors is associated with reduced colonic epithelial cell E-cadherin, enhanced epithelial cell IL-6 and Stat3 activation as well as crypt epithelial cell proliferation (197). These observations have generated significant interest over the last few years in how bacterial biofilms facilitate CRC development.

To confirm that the human biofilms are indeed carcinogenic, three murine models of carcinogenesis were evaluated by Tomkovich et al.: germ-free *Apc*^*MinΔ*850/+^ (129SvEv) mice, (b) germ-free *Apc*^*MinΔ*850/+^ *Il10*^−/−^ (129SvEv) mice, and conventional *Apc*^*MinΔ*716/+^ (C57BL/6) mice. In all three models, inocula prepared from biofilm-covered human mucosa induced colon tumors primarily in the distal colon at 12 weeks after inoculation. In contrast, inocula prepared from biofilm-negative mucosa did not (198). This study suggested that the biofilm itself might contribute to CRC pathology. Facultative anaerobic pathogens and pathobiont strains thrive in an inflammatory environment due to their ability to utilize inflammation-derived molecules such as nitrites and oxides as electron acceptors (199). In addition, microbial metabolism is altered under dysbiotic conditions, conferring new microbial phenotypes such as enhanced cellular adherence and invasion, mucus utilization, and production of metabolites and toxins (200). Microbiome profiling revealed that biofilms of distinct commensal bacteria, including *Bacteriodes fragilis, Fusobacterium* spp., and *E. coli*, were enriched in the CRC tumors, and these bacteria were able to promote CRC tumor development in genetically predisposed animals (184, 201–203).

Studies in murine models also showed that enterotoxigenic *B. fragilis* (ETBF) and colibactin-producing *E. coli* (CPEC) secrete carcinogenic toxins that are associated with the propagation of tumors (204–208). Furthermore, CPEC and ETBF were detected in patients with familial adenomatous polyposis, a premalignant disease that can develop into CRC (209).

*Fusobacterium nucleatum* accelerates tumorigenesis by inducing oncogenic and inflammatory responses in the gut through the production of the adhesin molecule FadA (210). However, whether FadA has toxigenic properties similar to those of colibactin or *B. fragilis* toxin is currently unknown. Finally, another enteric bacterium, *Campylobacter jejuni*, produces a genotoxin called cytolethal descending toxin (CDT) that induces colorectal cancer and changes in microbial composition and transcriptomic responses. Germ-free *Apc*^*Min*/+^ mice colonized with human clinical isolates of *C. jejuni* developed significantly more and larger tumors than the uncolonized mice or mice colonized with the *cdtB* mutant (211). Overall, these studies indicate a role for biofilm and toxin production by certain bacterial species as a driver for CRC pathogenesis.

It is plausible that toxins contribute to the onset of cancer by acting as direct environmental stressors. Toxins may also cause host cell DNA alterations. In germ-free mice that receive fresh feces from CRC patients, colon epithelia is renewed, more precancerous lesions are observed, and there is increased tissue and blood DNA methylation in intestinal tissues (212); this does not occur in mice given feces from healthy controls.

Recent studies showed a striking association between specific host microbes and aberrant DNA methylation in CRC. Addition or removal of acetyl and methyl residues at specific histone regions led to a corresponding gain or loss of DNA methylation at CpG dinucleotides, which led to an altered epigenomic state. CRC tumors can be grouped by CpG island methylator phenotype (CIMP); high CIMP, low CIMP or no CIMP (213). In CRC tumors where *Fusobacterium* species were substantially enriched, the tumors had a unique genetic and epigenetic profiles. The epigenetic changes were associated with high CIMP events and somatic mutations (213–215). However, additional studies are needed to determine whether bacterial toxins or whether certain bacterial species can directly promote oncolytic events by causing changes in DNA methylation. Additionally, dietary and digestive factors that are metabolized by microbiota can cause changes in the metabolic landscape and can alter the immune cell function (216). For instance, some short-chain fatty acids such as butyrate have anti-inflammatory properties and protect the host against colitis by increasing the level of colonic regulatory T cells and change the metabolism of epithelium (217–219). Therefore, dysbiosis of microbiota and localized enrichment of bacterial species may also directly influence the immune metabolism and function acting as a promoter or suppressor of tumor oncogenesis.

**Figure 3:**
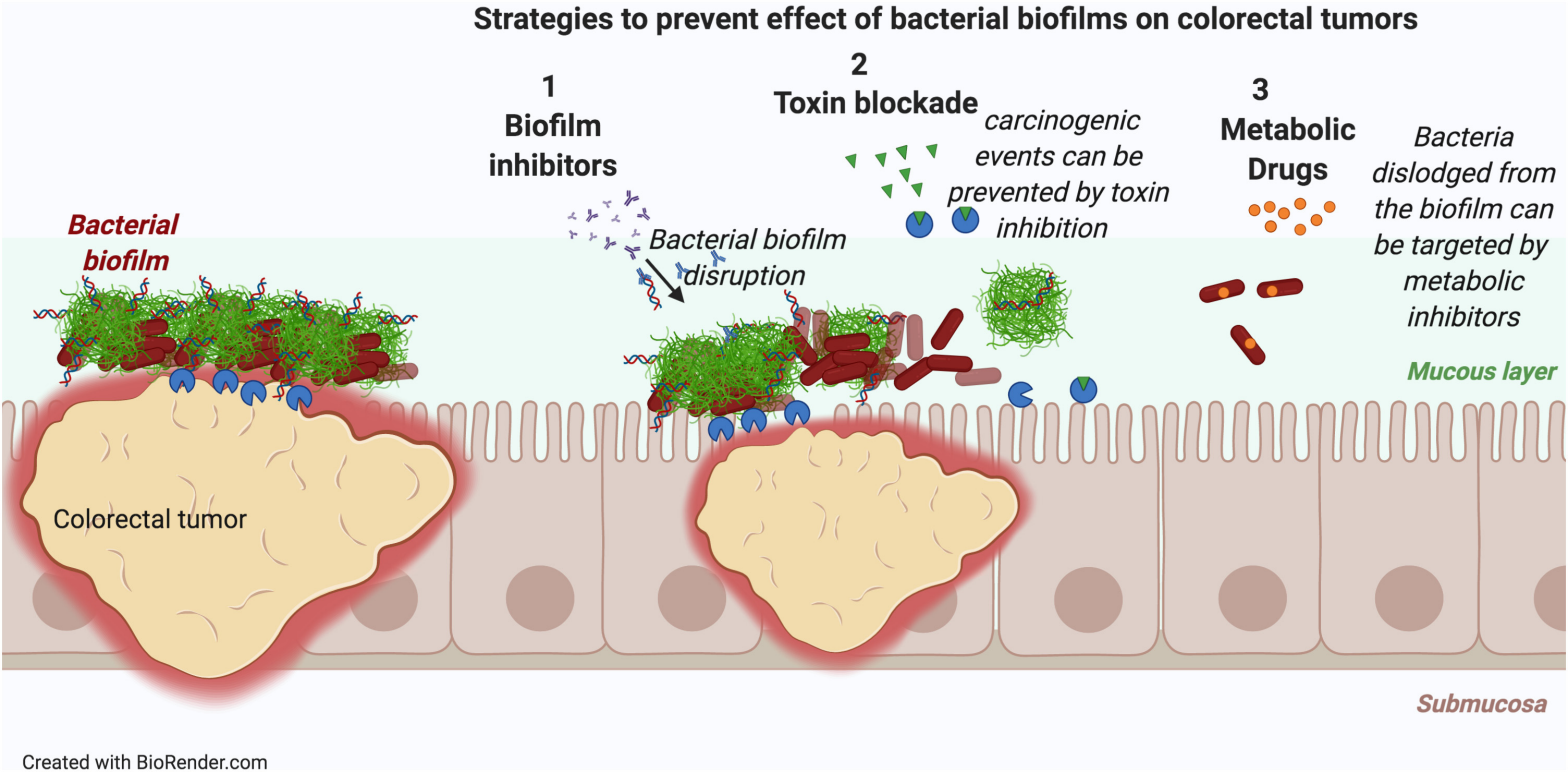
Novel strategies to intercept colorectal cancer. Three novel strategies either alone or in combination could reduce carcinogenesis triggered in biofilm associated colorectal tumors: (i) Inhibition of biofilm formation to dissociate the biofilms from the tumors, (ii) inhibition of toxin production by biofilm forming bacteria, (iii) inhibition of biofilm-associated bacterial growth by metabolic inhibitors.

These data suggest that restricting the biofilm-mediated colonization by toxin-producing bacteria and reversing the dysbiosis would be a first step to reduce the tumorigenesis. Three strategies are feasible: (i) inhibition of biofilm formation, (ii) inhibition of toxin production or activity, or (iii) targeted inhibition of tumor-associated bacterial growth (Figure 3). Consistent with the idea that inhibition of toxin production is a viable strategy, toxin-negative mutants of CPEC and ETBF do not elicit tumor formation in the azoxymethane/dextran sulfate sodium model of CRC (220, 221). Suppression of tumorigenesis has also been achieved by using a small-molecule inhibitor that directly targets the toxin colibactin (222). In support of utility of targeted inhibition of the toxin-producing bacteria, when the growth of CPEC and other *Enterobacteriaceae* are inhibited with the oral administration of sodium tungstate, a metabolic inhibitor that targets molybdoenzymes, development of malignancies was blocked (Figure 3) (223).

## Conclusions and Outlook

In the past decade, it has become increasingly apparent that the gut microbiota and infections with bacterial pathogens profoundly impact complex human diseases. In this review, we highlighted novel findings that indicate that biofilm-forming bacteria that produce the amyloid curli in the gastrointestinal tract are linked to autoimmune diseases, neurodegenerative diseases, and CRC. In healthy individuals, bacterial biofilms occupy the gut, and the extracellular matrix material educates the immune system to reinforce the epithelial barrier, preventing the leakage of bacterial ligands including pathogenic amyloids. However, the recent studies suggest that biofilms that harbor amyloid proteins like curli can initiate or accelerate pathogenic processes in a number of human diseases. The emerging picture suggests that in individuals that are genetically susceptible to chronic diseases, bacterial amyloids have pathogenic effects. For instance, in individuals who carry genetic risk factors such as HLA-B27, infections with invasive *Salmonella*, a pathogen that expresses curli, triggers autoimmunity and joint inflammation. Furthermore, enteric infections such as those with uropathogenic *Escherichia coli* trigger disease flares in SLE patients. It is important to note that the translocation of curli or curli-expressing bacteria into tissues is critical for the generation of autoimmune responses; this indicates that leakage of other amyloids or amyloid-producing bacteria from the gut or invasive infections may trigger similar responses. Consistent with this idea, infections with curli-producing enteric bacteria and other invasive bacteria that express amyloids, such as *B. burgdorferi, S. aureus, Pseudomonas* spp, and *M. tuberculosis*, trigger autoimmune reactions primarily affecting the joints (e.g., arthritis, septic arthritis).

Amyloid proteins are pathogenic in neurodegenerative diseases, and it was anticipated that bacterial amyloids like curli would influence aggregation of human amyloids like Aβ and αSyn. As predicted, recent work has shown that the colonization of the gut microbiota with curli-expressing *E. coli* increases αSyn pathology in mice that are predisposed to develop PD. However, whether curli can directly seed αSyn or whether it indirectly causes neuroinflammation and subsequent neurodegeneration is not known. Given these findings, treatments that reduce the pathological amyloid content in the microbiome or that reverse bacterial amyloid-induced neuroinflammation have potential as treatments for AD and PD.

One of the groundbreaking findings of the past several years was the demonstration that bacterial biofilms are associated with colonic tumors in humans and in animal models of CRC. Carcinogenic toxins produced by bacteria have been identified, but our current understanding of the direct effects of biofilms on the initiation and progression of CRC is limited. Given that we know how toxic amyloid intermediates form during initial stages of biofilm establishment, further work will be required to illuminate how curli or other amyloids contained in biofilms contribute to the onset of CRC by acting as a direct environmental stressor.

## Author Contributions

All authors listed have made a substantial, direct and intellectual contribution to the work, and approved it for publication.

## Conflict of Interest

The authors declare that the research was conducted in the absence of any commercial or financial relationships that could be construed as a potential conflict of interest.
